# New therapeutic approach to heart failure due to myocardial infarction based on targeting growth hormone-releasing hormone receptor

**DOI:** 10.18632/oncotarget.3303

**Published:** 2015-03-14

**Authors:** Rosemeire M. Kanashiro-Takeuchi, Luca Szalontay, Andrew V. Schally, Lauro M. Takeuchi, Petra Popovics, Miklos Jaszberenyi, Irving Vidaurre, Marta Zarandi, Ren-Zhi Cai, Norman L. Block, Joshua M. Hare, Ferenc G. Rick

**Affiliations:** ^1^ Interdisciplinary Stem Cell Institute, University of Miami, Miller School of Medicine, Miami, Florida, United States of America; ^2^ Department of Molecular and Cellular Pharmacology, University of Miami, Miller School of Medicine, Florida, United States of America; ^3^ Veterans Affairs Medical Center and South Florida Veterans Affairs Foundation for Research and Education, Miami, Florida, United States of America; ^4^ Department of Pathology, University of Miami, Miller School of Medicine, Miami, Florida, United States of America; ^5^ Department of Medicine, Divisions of Hematology/Oncology and Endocrinology, University of Miami, Miller School of Medicine, Miami, Florida, United States of America; ^6^ Sylvester Comprehensive Cancer Center, University of Miami, Miller School of Medicine, Miami, Florida, United States of America; ^7^ Department of Medicine III, Medical Faculty Carl Gustav Carus, TU Dresden, Germany; ^8^ Department of Medicine, Division of Cardiology, University of Miami, Miller School of Medicine, Miami, Florida, United States of America; ^9^ Department of Urology, Herbert Wertheim College of Medicine, Florida International University, Miami, Florida, United States of America

**Keywords:** growth hormone-releasing hormone, myocardial infarction, heart failure, remodeling, cardioprotection

## Abstract

**Background:**

We previously showed that growth hormone-releasing hormone (GHRH) agonists are cardioprotective following myocardial infarction (MI). Here, our aim was to evaluate the *in vitro* and *in vivo* activities of highly potent new GHRH agonists, and elucidate their mechanisms of action in promoting cardiac repair.

**Methods and Results:**

H9c2 cells were cultured in serum-free medium, mimicking nutritional deprivation. GHRH agonists decreased calcium influx and significantly improved cell survival. Rats with cardiac infarction were treated with GHRH agonists or placebo for four weeks. MI size was reduced by selected GHRH agonists (JI-38, MR-356, MR-409); this accompanied an increased number of cardiac c-kit^+^ cells, cellular mitotic divisions, and vascular density. One week post-MI, MR-409 significantly reduced plasma levels of IL-2, IL-6, IL-10 and TNF-α compared to placebo. Gene expression studies revealed favorable outcomes of MR-409 treatment partially result from inhibitory activity on pro-apoptotic molecules and pro-fibrotic systems, and by elevation of bone morphogenetic proteins.

**Conclusions:**

Treatment with GHRH agonists appears to reduce the inflammatory responses post-MI and may consequently improve mechanisms of healing and cardiac remod eling by regulating pathways involved in fibrosis, apoptosis and cardiac repair. Patients with cardiac dysfunction could benefit from treatment with novel GHRH agonists.

## INTRODUCTION

Current management of congestive heart failure (HF) serves to maximize the effectiveness of the remaining heart tissue in order to maintain an adequate cardiac output. However, even with the most advanced medical therapies for HF, morbidity and mortality remain high. Moreover, the susceptibility of the heart to injury and disease progression are markedly increased in advanced age. Consistent with the report from the American Heart Association Statistics Committee and Stroke Statistics Subcommittee, the incidence of HF per year rises to ~10 per 1000 population after 65 years of age [1]. Therefore, novel and innovative therapeutic approaches are urgently needed.

Besides the well-known renin-angiotensin-aldosterone system, other mechanisms play an important role in the pathophysiology of HF, including those based on vasopressin, natriuretic peptides, endothelin, and TNF-α [2]. Secretory events which occur in cardiomyocytes and fibroblasts play a critical role in the progression of myocardial remodeling leading to heart failure [3]. Both of these cell types secrete a variety of growth factors, cytokines, and hormones that influence cardiomyocyte growth and fibroblast activation in paracrine and autocrine manners. Our group reported recently that receptors for growth hormone-releasing hormone (GHRH-R) are present on the cell membranes of both cardiomyocytes [4, 5] and fibroblasts [6], and mediate the direct effects of this hormone. GHRH itself functions as an autocrine/paracrine growth factor in benign conditions [7–15] and various malignancies [16–22]. In addition, agonists of GHRH improve the survival and growth of many cell types such as pancreatic β cells [31] and cardiac stem cells (CSCs) [23]. GHRH has also been shown to stimulate proliferation and migration of mouse embryonic fibroblasts *in vitro* and to accelerate wound healing *in vivo* [6].

Recently, Granata *et al*. found that GHRH promotes cardiomyocyte survival and protects against ischemia/reperfusion injury in the isolated rat heart [24]. Our group has reported that after an experimental MI in rats, treatment with the GHRH agonist, JI-38, significantly decreased the degree of subsequent cardiac remodeling and dysfunction, suggesting a regenerative process [4, 5]. Most recently, boosting the cardiac reparative capacity by improving the intrinsic healing process has been increasingly considered as a novel therapeutic approach to complement repair deficiencies [25–27]. Accordingly, our group synthesized and investigated for their biological activity and cardioprotective effects additional new and more potent GHRH analogs. By incorporating non-natural amino acid substitutions into these synthetic analogs, we have increased their resistance to degradation, and their physiologic half-life. Thus the new GHRH agonists have significantly improved endocrine activity and stability making these agonists even more suitable for use in clinical settings [28]. The aim of the current study was to evaluate the *in vitro* and *in vivo* activities of these highly potent new GHRH analogs, and elucidate their mechanisms of action in promoting and/or enhancing cardiac repair.

## RESULTS

### Presence of GHRH ligand and GHRH receptor in H9c2 rat cardiomyoblast cell line

**Figure 1:**
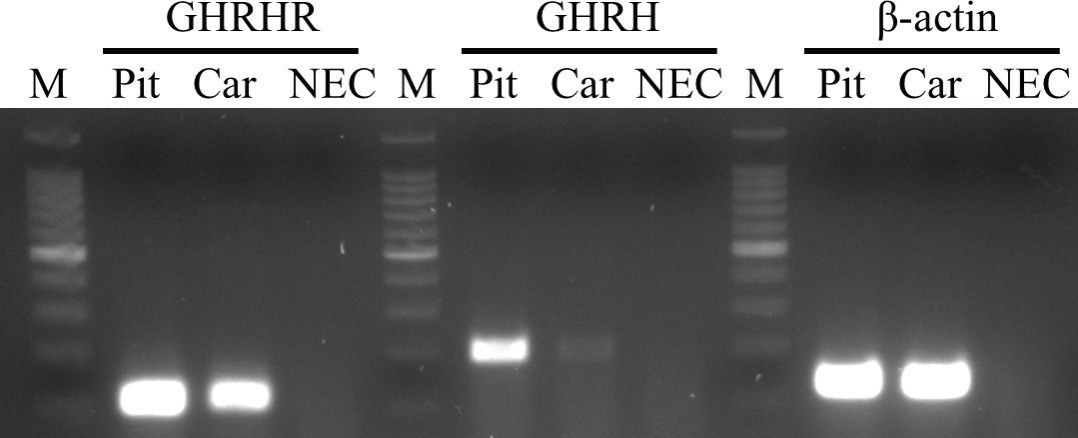
Expression of GHRH-R and GHRH mRNA in H9c2 cardiomyoblast cell line The PCR products of GHRH-R, GHRH and β-actin were detected at their expected sizes: at 110 bp, 195 bp and 133 bp, respectively. Abbreviations: Pit: rat pituitary, Car: H9c2 cells, NEC: no enzyme (reverse transcriptase) control, M: marker.

Reverse-transcribed mRNA from H9c2 cells and rat pituitary was subjected to RT-PCR. The amplicons for GHRH (195 bp), GHRH-R (110 bp), and β-actin (133 bp) were detected at their expected sizes (Figure 1).

### Effect of GHRH agonists on H9c2 cell survival in a nutritionally deprived environment

**Figure 2:**
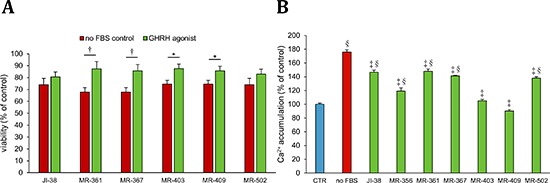
Effect of GHRH agonists at 100 nmol/L concentration on A. survival, and at 1 μmol/L concentration on B. Ca++-influx in H9c2 cardiomyoblast cells cultured in a nutrition-deprived medium All values represent means ± SEM. *p < 0.05, †*p* < 0.01, ‡*p* < 0.001, vs. placebo positive control and §*p* < 0.001 vs. vehicle control.

H9c2 cells were cultured in a nutritionally deprived environment for 72 hours, their media containing various GHRH-agonists at 100 nmol/L concentration. Cells in a serum free medium without any hormonal additions served as controls. All the tested GHRH-agonists, except JI-38 and MR-502, significantly improved the viability of the cardiac cells after 72 hours of starvation, compared to their control. The survival of H9c2 cells was increased from 67.8% to 87.3% after the treatment with MR-361, from 67.8% to 85.8% with MR-367, from 74.5% to 87.6% with MR-403, and from 74.5% to 85.7% with MR-409 (Figure 2A).

### Effect of GHRH agonists on calcium mobilization in a nutritionally deprived environment

Calcium influx is associated with cell death, and increase in intracellular calcium indicates ensuing apoptosis and necrosis. H9c2 cells were kept in a serum free medium for 72 hours, while they were exposed to various GHRH agonists at 1 μmol/L concentrations. Cells cultured in a medium containing FBS served as negative control, and cardiac cells in a nutritionally deprived medium, without any treatment, were the positive control. When compared to the positive control, all the tested GHRH-agonists significantly decreased the calcium influx in the H9c2 cells, 175.6% increase in the positive control vs. 146.3%, 119.1%, 147.9%, 141.3%, 105.1%, 90.2%, and 137.9%, in the cells treated with JI-38, MR-356, MR-361, MR-367, MR-403, MR-409, and MR-502, respectively (Figure 2B). However, two of these analogs, MR-403 and MR-409, which almost completely inhibited calcium influx, showed no significant difference when compared to the negative control.

### Effect of GHRH agonists on the expression of genes responsible for signal transduction activation and inhibition in H9c2 cell line

We investigated the actions of GHRH and its signaling in H9c2 cell line to determine mechanisms responsible for the survival observed in the treated cells. The Rat Signal Transduction Pathway Finder PCR array used in our study provided a simple and sensitive tool for profiling the expression of 84 key genes responsible for signal transduction pathway activation or inhibition. We identified important functional molecules affected by treatment with the GHRH agonists and selected genes potentially related to cell death, senescence and cardiac remodeling. After treatment with MR-409 more than 20 genes in the H9c2 cells exhibited significant transcriptional change in mRNA expression relative to control, and also relative to cells cultured in a nutritionally deprived environment without exposure to GHRH agonists (Table 1).

**Table 1:** Relative expression of genes related to cardiac remodeling in H9c2 rat cardiomyoblast cells after treatment with GHRH agonist, MR-409

Gene	Fold change
Axin-2	− 8.11
Bcl-2 binding component 3	− 3.53
Bcl-2 related protein A1d	− 14.2
Bone morphogenetic protein 2	4.53
Bone morphogenetic protein 4	5.98
Cyclin dependent kinase inhibitor 1A	− 4.99
Fos-like antigen 1	− 7.57
Inhibitor of DNA binding 1	− 6.15
Serpine 1	− 4.66

Modulated genes with at least 3-fold change relative to the untreated control after treatment of H9c2 cells with the GHRH agonist MR-409.

Importantly, expression of axin-2, cyclin dependent kinase inhibitor 1A (Cdkn1A), fos-like antigen 1 (Fosl1) and inhibitor of DNA binding 1 (Id1) genes were markedly downregulated (−8.11, −4.99, −7.57 and −6.15 fold, respectively) by MR-409 (Table 1). Similarly, transcription of genes encoding pro-apoptotic proteins, including Bcl-2 binding component 3 and Bcl-2 related protein A1d, was decreased (−3.53 and −14.2 fold, respectively) compared to the control group. We demonstrated a significant increase (4.53 and 5.98 fold, respectively) in the transcription of genes of bone morphometric proteins 2 and 4 (BMP2 and BMP4), respectively, in response to MR-409 treatment (Table 1). In addition, MR-409 decreased transcription of Serpine-1 gene, which encodes the plasminogen activator inhibitor-1 (PAI-1), a protein which is involved in extracellular matrix (ECM) synthesis and remodeling.

### Impact of GHRH analogs on myocardium infarct burden, scar thickness and vessel formation

To explore the mechanism by which agonists of GHRH improve cardiac performance following acute MI [4, 28], we selected three GHRH agonists (JI-38, MR-356 and MR-409) and examined their relationship to proliferative capacity of myocytes, vascular density and infarct size.

**Figure 3:**
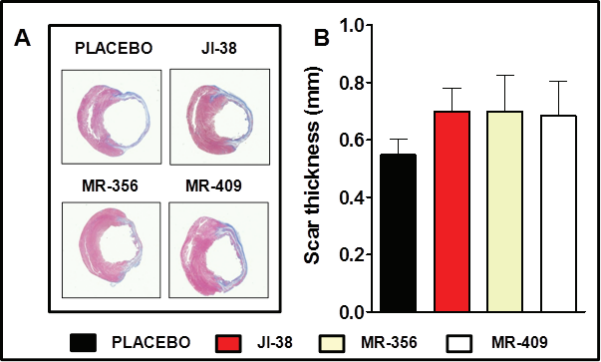
Impact of GHRH agonists on myocardium infarct burden and scar thickness **A.** Representative images of Masson-Trichrome staining showing reduced infarct size and presence of viable tissue in the scar area of the treated hearts. (**B.** Bar graphs correspond to scar thickness. All values represent means ± SEM (*n* = 4–6).

Infarct size was determined by morphometric measurements [29]. Our previous results [28] showed that size of the MI was substantially reduced by GHRH-agonists, JI-38 (42 ± 1%), MR-356 (37 ± 2.5%) and MR-409 (42 ± 2.4%) vs. placebo (49 ± 2%, *p* < 0.05 for all) confirming the agonists' cardioprotective effect. In addition, most of the hearts treated with GHRH analogs showed presence of viable myocardium in the scar area in contrast to the untreated hearts; in general, the average infarct wall thickness in the mid ventricle tended to be thicker than in the placebo group (Figure 3A and 3B).

**Figure 4:**
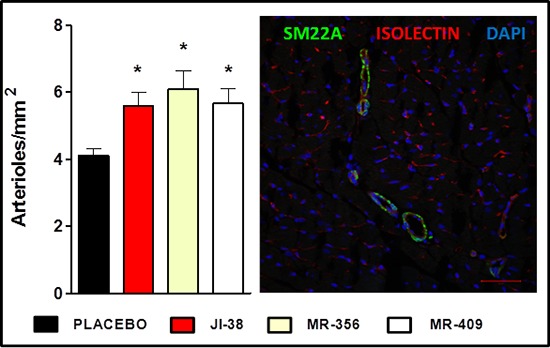
Effect of therapy with GHRH agonists on vessel formation **A.** Bar graphs represent the number of arterioles per mm^2^. All values represent means ± SEM (*p < 0.05 vs. placebo, *n* = 4–6). **B.** Confocal immunofluorescent image of smooth muscle 22 alpha (SM22A, green), isolectin B4 (red) and nuclei (DAPI, blue) staining. Scale bar: 50 μm.

Next combined isolectin and smooth muscle 22 alpha (SM22A) staining was used to identify arterioles (Figure 4), since they are of obvious importance for myocardium perfusion and oxygen supply. All animals treated with GHRH analogs showed higher vascular density (*p* < 0.05 vs. placebo). We also observed an inverse correlation between infarct size and vascular density (*p* = 0.0002, *r* = −0.7562).

### Effect of GHRH agonists on the magnitude of cardiomyocyte turnover and on cell proliferation

**Figure 5:**
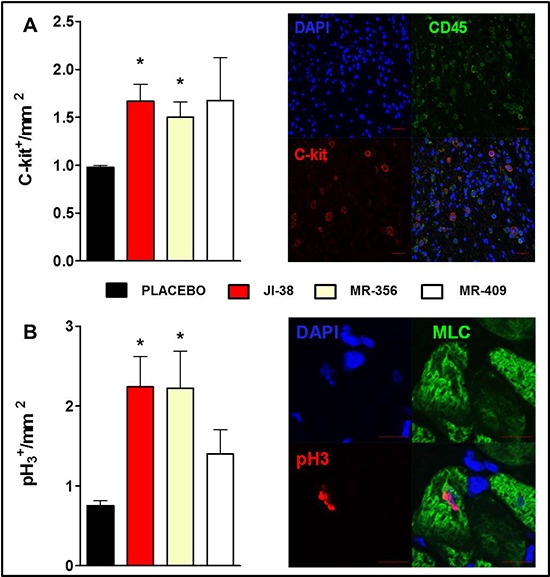
Effect of GHRH agonists on cardiomyocyte turnover and cell proliferation Immunostaining analysis of c-kit expression **A.** and cardiomyocyte mitosis **B.** based on the nuclear expression of phospho-histone H_3_ (pH_3_). Bar graphs show expression of c-kit^+^ cells and pH3^+^ cells per mm^2^ (*p < 0.05 vs. placebo, *n* = 5–6), respectively. Representative confocal immunofluorescent images illustrating expression of c-kit (red), CD45 (green) and nuclei (DAPI, blue) on the top panel (Scale bar: 20 μm) and co-localization of pH_3_ (magenta) and myosin light chain (MLC, green) and nuclei (DAPI, blue) on the bottom panel (Scale bar: 10 μm).

Cardiomyocyte mitotic division and c-kit expression were assessed by immunofluorescence. Treatment with GHRH analogs increased the number of endogenous cardiac c-kit^+^ cells and the cellular mitotic division (mitotic marker pH_3_) in the myocardium (Figure 5A and 5B, respectively). Furthermore, an inverse correlation was identified between MI size and c-kit expression (*p* = 0.0084, *r* = −0.5471) and cardiomyocyte mitotic division (*p* = 0.0096, *r* = −0.5390).

### Effect of administration of GHRH on levels of inflammation-related cytokines post-MI

**Figure 6:**
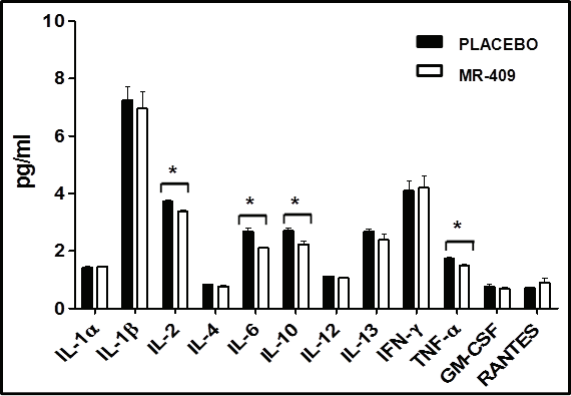
Impact of GHRH agonists on circulating cytokines post-MI One-week post-MI, treatment with MR-409 produced a significant reduction in plasma levels of IL-2, IL-6, IL-10 and TNF-α in comparison to the placebo group (*p < 0.05, *n* = 3 for each group).

Next, we investigated the action of GHRH agonist on the levels of inflammatory cytokines after MI (Figure 6). One week after starting the treatment with MR-409, there was a significant reduction in plasma IL-2, IL-6, IL-10 and TNF-α in comparison to placebo group (*p* < 0.05, *n* = 3 for each group).

### Effects of GHRH agonist therapy on the expression of genes related to ECM

**Figure 7:**
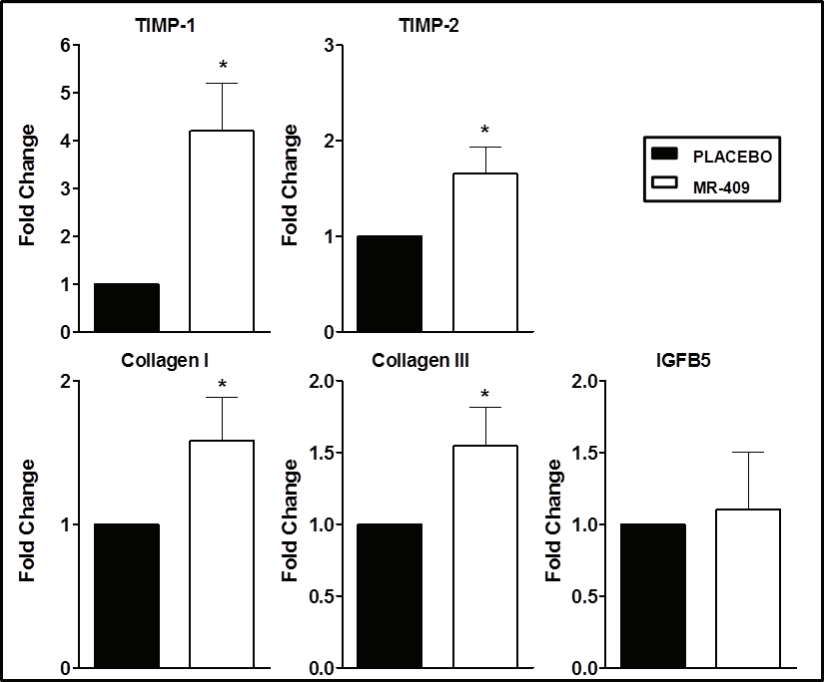
Effects of GHRH agonist therapy on the expression of genes involved in extracellular matrix (ECM) Hearts treated with MR-409 had markedly increased mRNA levels of TIMP-1, TIMP-2, and collagen I, and III (*p < 0.05 vs. placebo). There was no significant change in the expression of IGFB5. Results are displayed as average and standard error of the mean of triplicate samples (*n* = 3–4).

Relative gene expression levels of tissue inhibitor of metalloproteinase 1 (TIMP-1) and 2 (TIMP-2), collagen types I and III, and insulin-like growth factor binding protein 5 (IGFBP5) were assessed by RT-PCR in the placebo and in the MR-409 groups (Figure 7; Table 2). Hearts treated with MR-409 showed markedly increased expression of genes related to ECM (*p* < 0.05 vs. placebo), with the exception of IGFBP5 (*p* = ns).

**Table 2:** TaqMan^®^ probes used to examine the impact of treatment with GHRH analogs on mRNA levels of extracellular matrix (ECM) related genes

Gene	TaqMan^®^ probe
GAPDH	Rn99999916_s1
TIMP-1	Rn00587558_m1
TIMP-2	Rn00573232_m1
Collagen I	Rn01463848_m1
Collagen III	Rn01437681_m1
IGFBP5	Rn00563116_m1

## DISCUSSION

This work confirms and extends findings that GHRH agonists are cardioprotective following myocardial injury [4, 5, 28]. Consistent with our previous report, the reductions on MI size by GHRH agonists were accompanied by an increased number of resident cardiac c-kit^+^ cells, an increase in the number of cellular mitotic divisions in the myocardium, and also by increased vascular density in the myocardium.

In order to further investigate the mechanisms involved, we examined the levels of circulating inflammatory cytokines to elucidate the influence of GHRH treatment on LV remodeling. Normally, following MI, pro-inflammatory factors secreted by cardiac tissue, including cardiomyocytes and fibroblasts, may amplify cardiac injury and fibrosis [30]. Several factors are involved in the pathogenesis of maladaptive cardiac remodeling. Inflammatory reactions, including those mediated by cytokines, chemokines, and matrix metalloproteinases (MMPs) may play important roles in the progression of CHF. For instance, circulating levels of interleukin 6 (IL-6) are elevated after MI and are associated with increased morbidity and mortality [31]. Early inhibition of inflammatory cytokines in the treated group may thus preserve myocardial tissue, contribute to more effective tissue healing with reduced scar tissue formation, and thereby decrease mortality and morbidity. Importantly, our results demonstrate that administration of GHRH agonist, starting two hours after MI, reduces the levels of circulating inflammatory cytokines (IL2, IL-6 IL-10 and TNF-α) at one-week following MI. These findings suggest that GHRH-R activation can abolish the feedback loop of inflammation in the myocardium after ischemia.

We next examined the impact of treatment with these GHRH analogs on mRNA levels of ECM related genes. Our data show a substantial upregulation of genes related to the fibrosis pathway. The main action of TIMPs is to inhibit MMPs, but several groups have reported that TIMPs also have cell growth promoting, anti-apoptotic, steroidogenic and antiangiogenic activities [30, 32, 33], which are in part independent of their inhibition of MMP. Importantly, both TIMP-1 and TIMP-2 may activate the growth of fibrobasts *in vitro* [32–34], in addition to their MMP suppression. Thus, TIMPs may favor cardiac fibrosis not just by inhibiting MMPs, but also by separate direct pro-fibrotic mechanisms.

As the use of animals in experimental research is discouraged, H9c2, a clonal myogenic cell line derived from embryonic rat heart, has served as a surrogate for cardiac and skeletal cells and has become an important tool for toxicology [35] and signal transduction studies in the heart. Accordingly, our *in vitro* results show that treatment of H9c2 cells with GHRH agonists improved the cell viability in the presence of nutritionally deprived serum. The survival of the H9c2 cells with GHRH agonist treatment increased by an average of 22.02% when compared to the positive control. In addition, the starvation-induced increase in calcium influx was abolished by GHRH agonist treatment. Next, we investigated multiple signal transduction pathways involved on these effects.

Interestingly, our array data show that axin-2 (a classical target of the canonical Wnt pathway), a negative regulator of beta-catenin, was suppressed by MR-409, indicating modulation of the beta-catenin pathway. The exact role of beta-catenin in adult cardiac remodeling *in vivo* is still not understood; however, downregulation of beta-catenin is required for adaptive cardiac hypertrophy. mRNA levels of axin-2 were significantly increased in human fibrotic diseases and in murine experimental fibrosis [36]. Mastri *et al* showed that reduction of axin-2 expression, following downregulation of secreted Frizzled-related protein 2 (sFRP2) by mesenchymal stem cell therapy and sFRP2 antagonism by antibody blockage, counteracts the fibrogenic pathway and improves myocardial injury repair [37]. Accordingly, axin-2 appears to promote ECM accumulation and fibrosis through the Wnt-dependent pathway. Our present data thus suggest that beneficial effects of GHRH agonists on cardiac remodeling may involve suppression of Wnt signaling; however, further analysis is required to confirm this mechanism.

Noteworthy, MR-409 markedly suppressed the expression of Serpine 1. Overall, fibrosis in multiple tissues has been associated with increased expression of Serpine 1, also known as plasminogen activator inhibitor-1 (PAI-1) [38]. Serpine 1 is an inhibitor of plasmin action and was shown to be a target of TGF-β1, which implicates cross-talk between the members of the pro-fibrotic systems [39]. In addition, elevated levels of PAI-1 are associated with increased occurrence of thrombosis; and the decrease in its mRNA level may also protect against further ischemic events.

Recently, senescent cells are emerging as an important target in stem cell–based therapies, particularly, in the elderly population [40]. During the remodeling phase, senescent cells play a role in limiting scar formation by dissolving the fibrous proteins placed during the proliferative phase [41]. MR-409 limited the expression of Cdkn1A, an age-related gene tightly controlled by the p53 gene, which can modulate cell-cycle, differentiation, apoptosis, and senescence [42]. Torella *et al* [43] reported that downregulation of this gene can delay organ aging and heart dysfunction. On the other hand, Li *et al* [44] demonstrated that an increase in Cdkl1A expression occurs with an increase in apoptotic cells in rats with spontaneous heart failure.

The present findings also identified another factor related to age, Fosl1, a negative regulator of beta-catenin that plays an important role in limiting cell proliferation and differentiation [45]. Fosl1 has been characterized as a contributor to the AP-1 protein dimer complexes that regulate extracellular matrix, proliferation, and cell survival. In the presence of oxidative stress, FOSL1 can impair cell cycle entry or contribute to apoptosis.

Our data also show an overexpression of BMP2 and BMP4 genes. Accordingly, Wu *et al*. has reported that human recombinant BMP4 promotes survival in a cardiac muscle cell line after H_2_O_2_ injury, and also protects adult mouse cardiomyocytes against hypoxia-reoxygenation injury [46]. Therefore, this may be another one of the key mechanisms for the protection of the myocardium by the agonists.

In conclusion, activation of GHRH receptor in the heart by GHRH agonists may reduce the inflammatory response after MI and consequently improve the healing and cardiac remodeling by regulating pathways involved in fibrosis, apoptosis and mobilization of progenitor cells. This leads to significant recovery of the damaged myocardium and restitution of cardiac function. The reduction of MI size and increase in the number of mitotic cardiomyocytes provide evidence that administration of GHRH agonists enhances myocyte renewal and stimulates cardiac growth after MI, providing an effective therapeutic strategy to rescue/repair the infarcted heart. All three structural classes of the new compounds: methylamide analogs (MR-406 and MR-409), ethylamide analogs (MR-420 and MR-421) and C-terminal agmatine analogs (MR-356) are endowed with important endocrine and cardioprotective activities, and therefore such agonists could be useful for therapy of cardiovascular diseases, targeting specifically fibrogenic pathways.

## MATERIALS AND METHODS

### Peptides and reagents

In a search for more potent GHRH agonists, we synthesized, purified, and tested, both *in vitro* and *in vivo*, a series of new human GHRH(1–29) analogs. GHRH-agonists JI-38, MR-356, MR-361, MR-367, MR-403, MR-409, and MR-502 were synthesized by solid phase methods and purified by reversed-phase high-performance liquid chromatography as described previously [28]. The chemical structures listed in comparison with the older agonist, JI-38, are the following:

JI-38: [N-Dat^1^, Ala^2^, Phe^6^, Gln^8^, Orn^12^, Abu^15^, Orn^21^, Nle^27^, Asp^28^, Agm^29^]hGHRH(1–29)NH_2_
MR-356: [N-Me-Tyr^1^-JI-38]
MR-361: [N-Me-Tyr^1^, D-Ala^2^-JI-38]
MR-367: [N-Me-Tyr^1^, D-Ala^2^, Asn^8^-JI-38]
MR-403: [N-Me-Tyr^1^, D-Ala^2^, Arg^29^-NHCH_3_-JI-38]
MR-409: [N-Me-Tyr^1^, D-Ala^2^, Asn^8^, Arg^29^-NHCH_3_-JI-38]
MR-502: [D-Ala^2^, Phe(F)_5_^6^, Ser^28^, Arg^29^, Gab^30^-NH_2_-JI-38]

Noncoded amino acids and acyl groups are abbreviated as follows: Abu, alpha-aminobutyric acid; Agm, agmatine; Dat, des-amino-tyrosine; Phe(F)_5_, pentafluoro-phenylalanine; Gab, gamma-amino-butanoyl; hGHRH, human GHRH; Nle, norleucine; Orn, ornithine; N-Me-Tyr, N-methyl-tyrosine. The stability and purity of the compounds were checked by HPLC before the experiments. For *in vitro* studies, the peptides were dissolved in dimethyl-sulfoxide (DMSO) to final concentrations that did not exceed 0.1%. For *in vivo* studies, the peptides were dissolved in 0.1% DMSO in sterile aqueous 10% propylene glycol (vehicle).

### Cell line

Rat cardiomyoblast cell line, H9c2, was obtained from American Type Culture Collection (ATCC, Manassas, VA) and maintained in culture using Dulbecco's Modified Eagles's Medium (Invitrogen, Grand Island, NY) supplemented with 10% fetal bovine serum (FBS) and antibiotics (100 U/ml penicillin, 100 mg/ml streptomycin). Cells were grown at 37°C in a humidified 95% air/5% CO_2_ atmosphere.

### Cell survival assay (MTS assay)

Cells (10^4^/well) were seeded onto 96-well plates in 100 μl of culture medium containing 10% FBS, cultured overnight, then starved for 72 hours in a medium without fetal bovine serum (FBS). Cells in starving medium were exposed to hGHRH(1–29)NH_2_, or one of the GHRH agonists JI-38, MR-356, MR-361, MR-367, MR-403, MR-409, or MR-502, at a concentration of 100 nmol/L. Cell viability was evaluated by using a 3-(4, 5-dimethylthiazol-2-yl)-5-(3-carboxymethoxyphenyl)-2-(4-sulfonphenyl)-2H-tetrazolium (MTS) assay (CellTiter 96 Aqueous 1 Solution Cell Proliferation Assay; Promega, Madison, WI) according to the manufacturer's instructions. Absorbance was measured at 490 nm in a Victor 3 Multilabel Counter (Perkin-Elmer, Waltham, MD). All experiments were done in quadruplicate and were repeated 3 times. The inhibition of cell proliferation was expressed as the percentage of vehicle control (0.1% DMSO in the culture medium).

### Fluorescent assay for calcium mobilization

Cells (10^4^/well) were seeded onto 96-well plates with 100 μl of culture medium containing 10% FBS, cultured overnight, then starved for 72 hours in medium without FBS. Cells in starving medium were exposed to hGHRH, or one of the GHRH agonists JI-38, MR-356, MR-361, MR-367, MR-403, MR-409, or MR-502 at a concentration of 1 μmol/L. Calcium mobilization was evaluated by using (Fluo-Forte^®^ Calcium Assay Kits, Enzo Life Sciences, Framingdale, NY) according to the manufacturer's instructions. Fluorescence intensity was measured at excitation and emission wavelengths of 485 nm and 535 nm, respectively, with a Victor 3 Multilabel Counter. Intracellular calcium mobilization was expressed as a percentage of vehicle control (0.1% DMSO in the culture medium).

### Animal model

Recently, our group reported the endocrine and cardioprotective activities of several GHRH agonists [33]. Based on our findings, we selected tissues from three groups with enhanced activities in the heart for further analysis. Plasma (obtained at 1 week post-MI) and basal portions of hearts, free of fibrotic tissue, were flash-frozen in liquid nitrogen for total RNA isolation. Remaining tissue was fixed in 10% formalin (EMD Millipore, Billerica, MA) for histology. All animal procedures were carried out in accordance with the Guide for the Care and Use of Laboratory Animals (National Institutes of Health, revised 2011) and approved by the University of Miami Institutional Animal Care and Use Committee.

### Total RNA isolation and Reverse Transcription-Polymerase Chain Reaction (RT-PCR)

The expression of full-length pituitary GHRH receptor (pGHRH-R) and GHRH was detected by RT-PCR. Total RNA was isolated using NucleoSpin kit (Macherey-Nagel Inc., Bethlehem, PA). The yield and quality of total RNA was determined spectrophotometrically using 260 nm and 260/280 nm ratio, respectively. Genomic DNA was eliminated using Turbo DNA-free kit (Life Technologies, Carlsbad, CA). One and a half μg of RNA was reverse transcribed into cDNA with the RevertAid H minus RT Kit (Thermo Fisher Scientific, Waltham, MA) using a VeritiTM 96-well thermal cycler (Applied Biosystems, Foster City, CA). We evaluated the mRNA expression of GHRH-R, GHRH, and β-actin by using the Go Taq Flexi DNA polymerase kit (Promega, Madison, WI) with primers and method described previously [4, 47]. Normal rat pituitary was used as positive control. A sample that contained DNAse digested RNA and no enzyme during reverse transcription was used as negative control for RT-PCR.

In addition, total RNA from the remote area of left ventricle was isolated by the TriZol method (Invitrogen, Carlsbad, CA) as previously described [16]. We determined the mRNA expression of genes involved in ECM. Samples tested were obtained from 3–5 independent experiments.

### Histology

Slides were prepared with H&E and Masson's trichrome stain to assess cardiac structure and the presence and extent of fibrosis and myocardial scar, respectively. The infarct size was determined by morphometric measurements calculated as previously described with minor modifications [35]. Briefly, MI size was calculated by dividing the perimeter of the infarcted wall by the total LV perimeter wall using Adobe Photoshop-CS3 (Adobe Systems Inc., San Jose, CA, USA). An average of four sections of each heart was used to measure the infarct size. To evaluate the scar thickness, we measured the scar at midventricular level, averaging 3 measurements along the infarct wall. All measurements were performed blindly (*n* = 4–6 for each group) to avoid sampling bias.

### Immunofluorescence staining

Heart sections were deparaffinized with xylene and rehydrated in alcohol series and water as previously described [4]. Omission of the primary antibodies on parallel sections was used as negative control. Nuclei were labeled with 4′,6-diamidino-2-phenylindole (DAPI, Invitrogen, Carlsbad, CA). The total numbers of positively-stained cells at midventricular level were quantified per slide to calculate the number of cells per square mm (mm^2^) on each sample. All images were obtained with a fluorescent microscope (Olympus IX81, Olympus America Inc., Center Valley, PA) or a LSM710 Zeiss confocal laser-scanning module (Carl Zeiss MicroImaging, Germany).

Dual fluorescence immunostaining for isolectin B4 conjugated to Alexa 568 (Invitrogen, Carlsbad, CA) and α-SM22A (rabbit polyclonal, Abcam, Cambridge, MA) followed by donkey anti-rabbit FITC (Jackson ImmunoResearch Laboratories, West Grove, PA) was carried out to quantify arteriole density. The whole heart section at midventricular level was used for counting vessel profiles and the vascular density was expressed as arterioles per square mm (mm^2^).

### Detection of inflammatory cytokines using ELISA

The panel of inflammatory cytokines was measured in plasma samples collected from control and MR-409 treated groups at 1 week after treatment (*n* = 3 for each group) using the rat cytokine Multi-analyte Elisarray kit (Qiagen Inc., Valencia, CA). The arrays were performed according to manufacturer's instructions. Absorbance was measured at 450 nm within 30 minutes of stopping the reaction. We measured production of the following cytokines: IL1α, IL1β, IL2, IL4, IL6, IL10, IL12, IL13, IFN-γ, TNF-α, granulocyte-macrophage colony stimulating factor (GM-CSF) and RANTES.

### Statistical analysis

For statistical evaluation, SigmaStat 3.0 software (Stat Software, San Jose, CA) or GraphPad Prism software (San Diego, CA, USA) version 5.0 for Windows was used. Results are expressed as mean ± SEM. One-way analysis of variance (ANOVA) followed by Bonferroni *t*-test or a two tailed Student's *t*-test were used where appropriate, and significance was accepted at *p* < 0.05. Correlations between MI size and vascular density, c-kit expression and cardiomyocyte mitotic division were tested by linear regression analysis.
